# Embracing Diversity, Equity, and Inclusion in the Scientific Community—Viewpoints of the Diversity, Equity, and Inclusion Committee of the North American Vascular Biology Organization

**DOI:** 10.3389/fcvm.2022.863256

**Published:** 2022-04-13

**Authors:** Mahdi Garelnabi, Mitzy Cowdin, Yun Fang, Bandana Shrestha, Masuko Ushio-Fukai, Elena Aikawa, Garth Graham, Grietje Molema, Hiromi Yanagisawa, Masanori Aikawa

**Affiliations:** ^1^Diversity, Equity, and Inclusion Committee, North American Vascular Biology Organization (NAVBO), Germantown, MD, United States; ^2^Department of Biomedical and Nutritional Sciences, University of Massachusetts, Lowell, MA, United States; ^3^University of Texas Southwestern Medical Center, Dallas, TX, United States; ^4^Section of Pulmonary and Critical Care, Department of Medicine, University of Chicago, Chicago, IL, United States; ^5^Medical College of Georgia at Augusta University, Augusta, GA, United States; ^6^Brigham and Women's Hospital, Harvard Medical School, Boston, MA, United States; ^7^Healthcare and Public Health Partnerships, YouTube and Google Health, Playa Vista, CA, United States; ^8^University Medical Center Groningen, Groningen, Netherlands; ^9^Life Science Center for Survival Dynamics, TARA, University of Tsukuba, Tsukuba, Ibaraki, Japan

**Keywords:** African American, Asian American, Latinx, social justice, Native American, STEM women, diversity and inclusion

## Abstract

Recent increased visibility on racial issues in the United States elicited public outcry and a collective call for action. The social justice movement has facilitated energetic discussions about race, sexual orientation, and various issues of diversity, equity, and inclusion. This article discusses issues faced by people of color that we as scientists can address, as well as challenges faced by women and internationally trained scientists in the scientific community that need immediate attention. Moreover, we highlight various ways to resolve such issues at both institutional and individual levels. Silence and incremental solutions are no longer acceptable to achieving lasting social justice and ensure prosperous societies that work for all.

## Introduction

Everyone deserves to live in dignity. However, reality is far from ideal, even in highly developed nations. Barriers to diversity, equity, and inclusion are major obstacles. The United States (US) recently faced an immediate need to address complex issues associated with the consequences of racism against people from the African-American and Asian-American communities. In addition to fundamental diversity issues, apathy may be another challenge. As the media's attention to racially charged events fades, attention shifts, particularly for those who are not directly affected. Lawmakers turn to other issues and potentially fail to act. Indeed, while the large-scale protests of summer 2020 have diminished, racial inequality and disparity remain inadequately unaddressed. Other diversity issues in the scientific community (e.g., gender, sexual orientation, culture, socioeconomic status, and religion) need equal attention. Recognizing such issues is only the first step, and international organizations, governments, institutes, and individuals must work to understand problems, educate each other, and find solutions. Professional organizations must adopt strict, zero tolerance policies toward any form of racism and inequality.

As scientists, we should act swiftly to find solutions. The North American Vascular Biology Organization (NAVBO) has built a strong tradition of embracing and promoting diversity. In response to the need for justice, NAVBO launched the new Diversity, Equity, and Inclusion Committee and issued a Statement of Commitment to Diversity, Equity, and Inclusion^1^,^2^. Here, the committee offers potential solutions to the various issues of diversity and inclusion the world and scientific communities face. Together, we can work to protect our core values and establish safe and compassionate environments.

## I: Overcoming Social Injustice and Moving Forward

### Racial Inequality Is Real and Can Affect Public Health

Recent elevated visibility on the issues of racial injustice in the US has elicited public protests and a collective call for action^3^, prompting us to increase our fight against prejudice, ignorance, and indifference. We hope these protests end all forms of discrimination, not only against African-, Native-, and Asian-American people but also against other races and ethnicities, gender or gender identity, sex or sexual orientation, age, religion, culture, beliefs, national origin, immigration status, language proficiency, socioeconomic status, or intellectual or physical ability^4^,^5^,^6^ (1). NAVBO's moral convictions cannot tolerate discrimination. The positive side of recent events is that they created an opportunity for people of all backgrounds to unite and form transformational movements in many institutions in the US and around the globe, leading to the development of policies and legal frames designed to uproot all forms of inequality in society at large.

Study after study, from all angles, clearly show that many institutions suffer from biases and discrimination against African Americans and other people of color (1–5). In the US, most African Americans (92%), Latinx (78%), Native Americans (75%), and Asian Americans (61%) report experiencing racial discrimination (i.e., racial slurs, violence, threats, and harassment) at work and in schools. Institutions that honestly assessed their own racial fairness discovered concerns of inequality. For instance, an institutional assessment of the American College of Physicians (ACP) reported that its membership applications considered race and religion, required citizenship in North America, and limited membership to English-language speakers in the first half of the 20th century (1). Gathering evidence from similar efforts by other groups would increase awareness of racial inequality. This is an important first step in establishing fair environments in the scientific community.

Racial injustice and disparity, including reduced access to employment, education, and crowded and substandard housing, affects public health and life expectancy in several ways. Discrimination causes adverse cognitive and emotional inadequacy, which can lead to lack of interest in healthy behaviors (e.g., sleep and exercise) and increased desire for unhealthy behaviors (e.g., alcoholism, drug abuse, and unhealthy food consumption) (6–8). Minorities have contracted COVID-19 far more often than the White populations. Recently published data show that African Americans disproportionately lost employment or income (9). Additionally, African Americans are more likely than White people to display vaccine hesitancy due to confidence and circumspection (10). As described by the Okorodudus, unfair treatment of young people by medical professionals may have led African Americans to distrust the entire medical system (11). Moreover, Albert et al. showed that health disparities are not merely differences in health status but rather an accumulated unfair practice in the healthcare system that can be modified for the well-being of the entire community (12). The social injustice and health disparities does not stop with African Americans, it is well documented in American Indian and Alaska Native people who have experienced health disparities when compared with other Americans. Lower life expectancy and disproportionate disease burden. They also experienced inadequate education, disproportionate poverty, discrimination in the delivery of health services, and cultural differences. All these issues are directly connected to socioeconomic difficulties (13).

### The Role of Educational Institutions in Promoting Social Justice

Institutions of higher learning are critical platforms for fostering equality and promoting social justice. Accessibility to these institutions by people of color represents an important socioeconomical tool for supporting their communities. Because a college degree can alter a family's trajectory for generations, scholarships and other resources are important to the success of students from underrepresented communities. Thus, institutions should increase their financial commitment to diversity as a means to retain and graduate students of color (14). Updated curricula that highlight the historical struggle of African Americans and offer restorative approaches to expose the past head-on can provide avenues for a brighter future. Training is crucial to the success of underrepresented students.

Importantly, faculty should mirror the student body because the success of African American and other faculty of color helps ensure the success of students from underrepresented communities (15, 16). Institutions should provide resources (e.g., adequate startup packages, mentorship, fair performance assessments) and tools that ensure the success of these faculty members. To retain faculty and trainees from underrepresented communities, institutional leadership must acknowledge and understand their experiences of racism (17). In addition, minority faculty require access to federal funding (2). Importantly, fair treatment by professional organizations helps ensure people of color that they can present their scientific work at conferences and symposia, chair scientific sessions, and compete for recognition (i.e., awards and recognitions), boosting their career and ensuring academic growth.

Professional organizations such as NAVBO should adopt strict policies and zero tolerance approaches to any form of racism or inequality, and work toward eliminating all forms of implicit bias in professional activities. Further, professional organizations must implement proactive methods to ensure fairness and support members of underrepresented communities. Dedicated committees that focus on programs and policies that promote diversity, equity, and inclusion can foster the success of faculty members from underrepresented communities. Some scientists, who do not understand how much underrepresented groups and minorities struggle, can unconsciously fall into implicit bias. Therefore, professional organizations must regularly conduct training programs and seminars on diversity and implicit biases and encourage scientists to increase diversity in their own institutions, research programs, and leadership positions. Notably, several studies indicate that diversity in the faculty or student body alone is not sufficient for resolving inequality. While many institutions have done a remarkable job diversifying their campuses, leadership often remains disproportionally dominated by white men (18).

**Figure 1:**
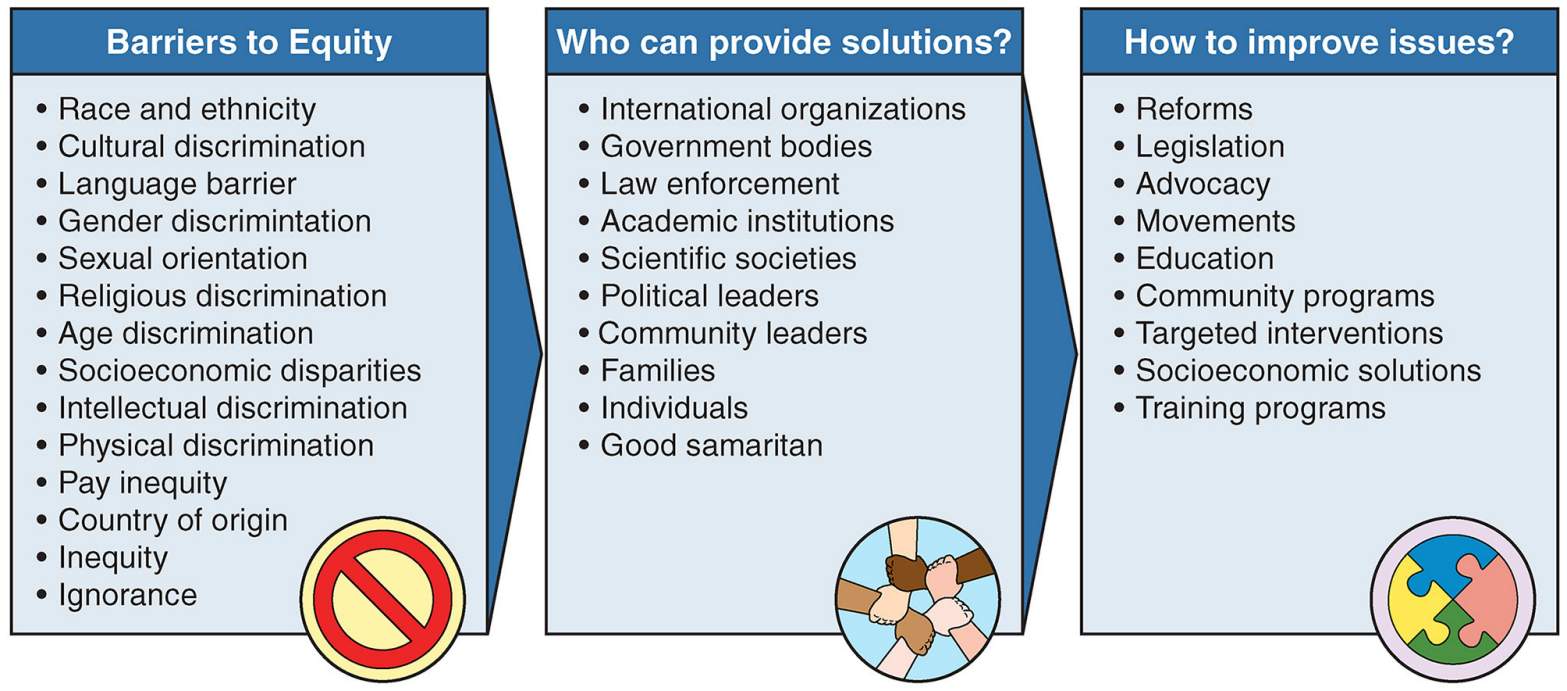
Barriers to equity: who can provide solutions and how?

We now recognize a greater need for reforming institutional practices in government, academia, sports, cultural societies, arts, etc. Inequality and injustice cannot be resolved without greater awareness, real efforts, and commitments from all sections of a society. It is not enough to merely issue statements that condemn injustice or simply post policies or statements on institutions' webpages. Our workforce should reflect the community. Identifying hidden discrimination in hiring, promotion, and access to resources is very important when implementing a fair workforce environment (Figure 1). Leaders, administrators, and educators of all backgrounds and at all levels need to act by adopting strategies with measurable outcomes.

### Role of the National Institutes of Health

The US National Institutes of Health (NIH) is the largest public funding agency of biomedical and behavioral research worldwide. Its mission is to seek fundamental knowledge about the nature and behavior of living systems and then apply that knowledge to enhance health, lengthen life, and reduce illness and disability. In response to recent events that highlighted the reality of racial injustice in the US, the NIH issued a statement that recognizes systemic racism in biomedical research and strengthened its commitments to end structural racism in the biomedical workforce. The statement also acknowledged its previous efforts have been insufficient^7^. The NIH is committed to developing new ways to support diversity, equity, and inclusion, and identifying and dismantling any policies and practices that may harm our workforce and our science (2).

The NIH recently launched UNITE, a new initiative that aims to identify and address structural racism within the NIH and the greater biomedical community^7^. Briefly, UNITE aims to establish an equitable culture within the biomedical research enterprise, reduce barriers to racial equity in its workforce, and advance health equity research to eliminate or lessen health disparities and inequities. UNITE also seeks to (i) identify critical elements that perpetuate structural racism and leads to a lack of diversity, equity, and inclusion in the biomedical enterprise; (ii) investigate and eliminate health disparities and inequities; (iii) change the organizational culture and structure of NIH to promote diversity, equity, and inclusion; (iv) coordinate NIH-wide efforts to ensure transparency, accountability, and sustainability of all UNITE efforts; and (v) evaluate NIH extramural policies and processes to identify and change cultures, practices, and structures to promote inclusivity and diversity within the extramural research ecosystem.

The NIH also promotes diversity, equity and inclusion by requiring the involvement of African American and other underrepresented people as presenters, a score-driving factor for R13 applications to support scientific conferences. This effort helps increase awareness of social injustice issues in science communities.

In addition to the NIH's efforts, collective action by all stakeholders (i.e., communities of scientific research, advocacy, clinical practice) and non-scientific communities, including the public, is crucial to ending systemic racism in the biomedical research workforce.

## II: Challenges in Career Development for Women

### The Underrepresentation of Women in Medicine Biological Sciences, and Science, Technology, Engineering, or Math

Although women's participation in biomedical sciences and other sciences, technology, engineering, and math (STEM) fields has increased, evidence suggests that women are still underrepresented in education, careers, and leadership positions (19). A large comprehensive bibliometric analysis of 1.5 million authors, led by systems scientist Albert-László Barabási, reported longitudinal gender gaps in academic publishing and significantly higher dropout rates in women (20). Particularly, women from racial and ethnic minority groups (e.g., African American, Hispanic, or indigenous) are insufficiently represented in various scientific professions and career levels. After graduating from high school, women are less likely than men to choose any of the STEM fields as a major in college. Women's representation in science decreases further at the graduate level, the transition to the workplace, and still further at senior and professorial levels^8^. Barriers to women's career progression partly explain their underrepresentation in faculty and leadership positions in medicine, biological sciences, and STEM. Importantly, women and men would share similar opportunities if the legal system supported them equally. Gender inequality should be recognized as a major social problem and a primary consideration for the members of our scientific community. A resolution of gender inequality will help improve women's education and careers and encourage more women to establish a career in science, benefitting society at large.

### Understanding Barriers to Women's Career Development in Science

Evidence suggests that higher dropout rates for women in academic careers compared to men (20). Why? Barriers to career development for women are institutional, cultural, individual, and family-bounded. Institutional barriers include (i) lower-level professional positions, (ii) lower pay, (iii) dysfunctional legislations that hinder promotion, (iv) negative attitudes, and (v) limited access to networks and mentors (21). Other barriers include gender stereotypes, discrimination and harassment, and gender-role expectations. Self-recognized stereotyping and common biases seem to be the first obstacles for female graduates who begin a career in STEM. Such misconceptions include: (i) men are better than women in STEM fields; (ii) women are not interested in careers in science; and (iii) successful women behave in masculine ways. Gender bias may exist in peer review, job applications, recruitment, and promotions (22, 23). Responsibility for childcare or caring for an ill or aging family member is another typical barrier to women's career development. Frequently, men and women do not share housework, childcare, and looking after the family's emotional well-being equally. Thus, many women scientists who strive to establish an independent career must balance work and family responsibilities, leading to career breaks or part-time appointments. A consequent reduction in publications has a negative impact on grant success rates and career development, resulting in job insecurity and reduced career retention.

### Strategies to Overcome Barriers to Career Progression for Women

Mentoring and role-modeling programs help women develop a network with multiple mentoring relationships for degree and career success, in STEM (24). Mentoring programs can strengthen women's motivation and persistence in career pathways through (i) providing female role models; (ii) teaching women how to grow their mentor–mentee network; and (iii) introducing women to local mentors and sponsors. Training for issues related to stereotypes and unconscious biases, along with transparent recruitment and promotion processes, are required. Detailed plans to promote cultural shifts can ensure that women have equal access to opportunities and adequate paths to develop their career in science. Since most research appointments depend on active, independent grants with short-term contracts, women's pathways for promotion are often unclear^9^,^10^.

Understanding a diversity-oriented working environment and identifying good mentors, collaborators, and peers can help women avoid isolation, stay connected with networks, and develop their careers in science. Organizations should train employees on unconscious bias, diversity, and inclusion to encourage shifts in culture and improve strategies to recruit and retain female scientists. In addition, policies should promote an inclusive workplace and foster transparent promotion processes that ensure pay equity.

When facing such issues, female scientists should not struggle alone. Our institutions have a key responsibility to create safe working conditions that enable all employees to deliver high-quality work in an effective manner. Such environments have no place for workplace bullying or any other form of harassment (25). All personnel need active support to make our academic and research institutes gender-balanced at all the levels, from technical or administrative staff to junior investigators to full professor, provost, or institute director. Simply stated, diverse organizations perform better (26).

Any organization that is seriously interested in expanding gender diversity can instantly implement a “Zipper quota,” a method coined by Curt Rice^11^. This method shortlists suitable women candidates and “zips” them together with a shortlist of suitable male candidates. Next, shortlisted candidates are selected by putting the first woman on the interview list, then the first man, followed by the 2nd woman, the 2nd man, and so on. Thus, any zipped list will yield a new shortlist for interviewing the best candidates. The strategy can be applied to recruitment for any position, and it provides recruitment committees with the exciting opportunity to interview the best female and best male candidates. In addition, the composition of recruitment committees can be established by “zipping” shortlists of its female and male members. This strategy minimizes the chances of selecting or promoting “the usual suspects;” instead, the best person is hired based on their own merits and the best scholars are promoted. Since women and men are equally smart and skilled, hiring qualified women will become part of the norm.

### Imposter Syndrome

Imposter syndrome (i.e., doubting your own abilities) often hinders women's success, and it can be generalized to any underrepresented group. Imposter syndrome internalizes thoughts that you are not good enough, do not belong, feel like a fraud, do not deserve the job or promotion. Additionally, you devalue your worth and think you have only succeeded due to luck, not because of your talent or qualifications. Imposter syndrome disproportionately affects minority groups and high-achieving women who struggle to accept their own accomplishments (25, 27). It persists throughout college and graduate school and into the working world, where women tend to judge their performance as worse than it is, while men judge their own performance as better. Even the most successful and accomplished women have this experience. Although imposter phenomenon is not an official diagnosis, psychologists and others acknowledge that it is a real and specific form of intellectual self-doubt. Imposter feelings generally include anxiety and depression, which greatly reduce confidence and cause women to consider leaving the field. Mentors who provide advice on how to deal with impostorism are especially helpful. Importantly for women, overcoming imposter syndrome involves recording their accomplishments; visualizing and recognizing their success; talking to mentors, colleagues, or friends; removing doubt; becoming confident; realizing that no one is perfect, and reminding themselves that they are good at what they do.

### Impact of COVID-19 on Women's Career Development in Science

COVID-19 continues to spread around the world with unexpected consequences for health systems and global economies. Compared to men in science, the pandemic has greatly affected disadvantaged women, particularly in STEM (28–32). Women from diverse backgrounds have additional barriers during the pandemic, including homeschooling their children, general housework, and managing their paid workload. Therefore, women are less likely than men to attend STEM workplaces, submit manuscripts and grant applications, and start new projects. Because COVID-19 has caused budgetary restraint at some universities and academic medical centers, STEM jobs are at risk. Significantly reduced short-term contracts are held mainly by women (28). We hope that pandemic-related budget cuts do not influence ongoing equity programs. The future of women in STEM could be jeopardized if developments achieved in recent years are lost. Solving this issue requires consideration and discussion of how to mitigate the impact of COVID-19 on job security and career progression for women in medicine, biological sciences, and STEM.

## III: LGBTQ+ in Stem

Although social science research has made great strides in documenting and reporting the problems that racial minorities and women face in STEM, other sociodemographic minorities (e.g., sexual and gender minorities) have received less attention. This article refers to such minorities including but not limited to gay, lesbian, transgender, queer, non-binary, and asexual groups as LGBTQ+ (sometimes LGBTQ, LGBQ, or LGBT+, depending on context). These minorities are interesting in the context of inequality, but sampling limitations have prevented investigation into systemic inequalities faced by LGBTQ+ scientists. Recent studies report the climate surrounding LGBTQ+ individuals in STEM as significantly less pleasant compared to their heterosexual counterparts (32, 33). Specifically, sexual minorities were 7% less likely to finish a STEM degree compared to switching to a non-STEM major, although sexual minorities were more likely to participate in undergraduate research programs (34). In the workplace, up to 28% of LGBT scientist respondents have considered leaving their STEM workplace; transgender individuals were hardest hit, with nearly half having considered leaving; and 20% of transgender respondents frequently considered leaving (34, 35). Forty-nine percent of respondents agreed that there is an overall lack of awareness of LGBT+ issues in STEM. Notably, however, 70% of respondents reported that the working environment for LGBT+ scientists was improving (35).

## IV: First Generation in STEM

**Figure 2:**
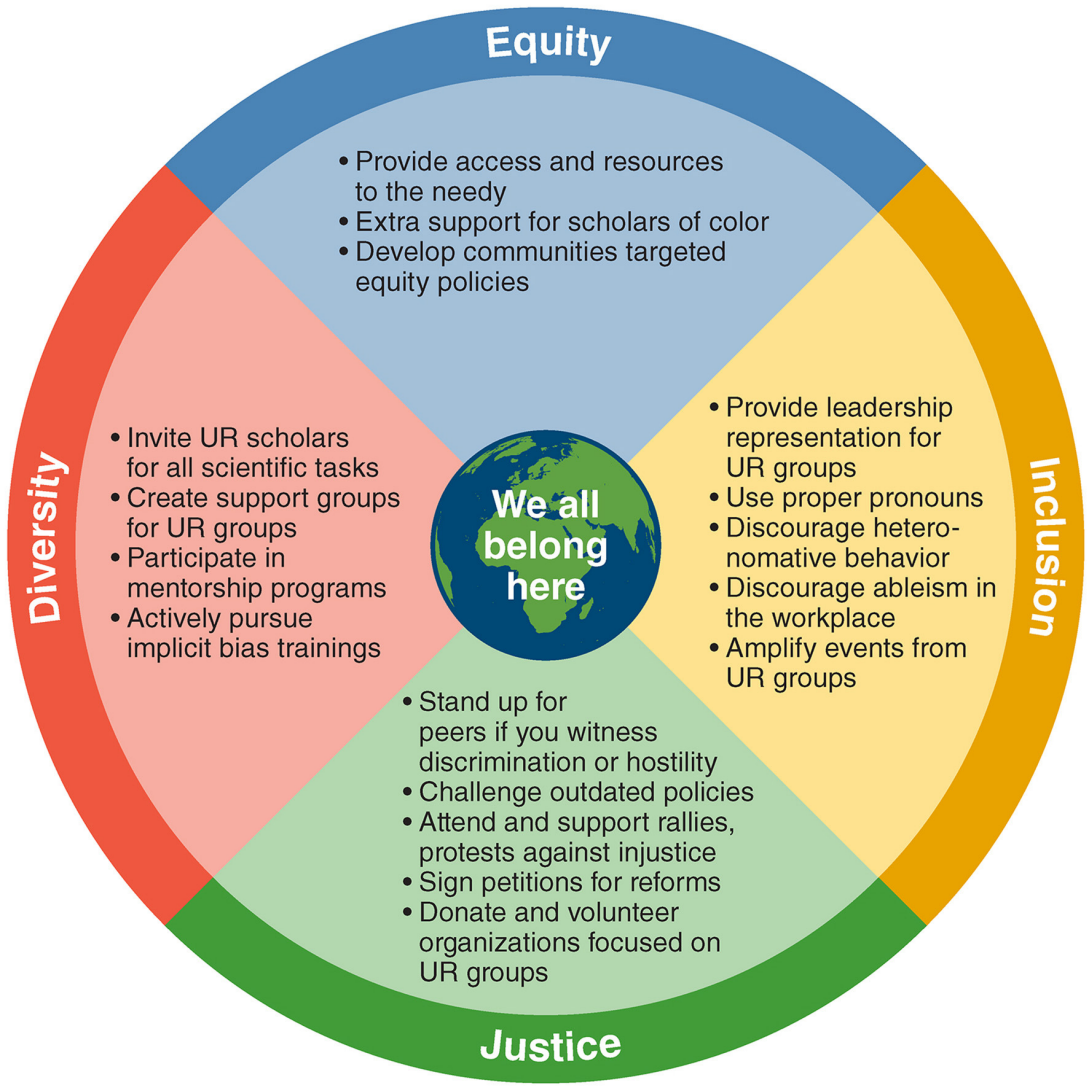
How can we be part of the solution.

Being a first-generation (“first-gen”) scientist comes with its own unique challenges. First-gen students are those whose parents did not acquire any higher education after high school or the equivalent (36). These students often do not know how to navigate the college application process, especially for graduate school. Disadvantages facing first-gen students include (i) inability to speak to their parents and/or guardians about college resources, (ii) navigating college applications, and (iii) applying for grants or scholarships. These disadvantages are compounded for first-gen students from underrepresented racial groups, who may experience discrimination, racism, and language barriers while dealing with the hurdles of the college application process (37). In fact, Latinx students comprise a significantly larger portion of first-gen students (38). First-gen students are less likely to pursue an undergraduate or graduate degree due to many factors, but notably many must work one or more jobs to pay for college. Therefore, first-gens cannot fully devote themselves to extracurricular activities and gain experience to pursue graduate education (Figure 2) (38–40).

## V: Challenges of Internationally Trained Scholars

### Barriers in Communication and Grant Writing

While this section focuses on how young international students can overcome difficulties and succeed, we also wish to increase awareness of the challenges foreign-born scientists experience in a new country, particularly the US. Discussing cultural differences and immigration restrictions can present fundamental challenges. Starting new careers as postdoctoral scientists and then moving into faculty positions in a new country are further compounded by cultural and language barriers. Although most postdocs and faculty members were educated in English and/or have acquired English language skills, developing effective communication skills across deep-rooted accents and vastly different cultural settings can be very difficult.

Alongside informal and social interactions, grant writing and presentation skills are critical to success in an academic career. Mastering such skills requires a great deal of practice and adaptation by foreign-trained scholars. When writing competitive grants, such scholars encounter differences in language and cultural communication styles, which often hinder the process and logical flow of ideas. Some languages, such as Japanese, are indirect and less clear compared with American-style English (e.g., the concept of “conclusion first”). Understanding differences in writing styles plays a substantial role in shaping grant applications, leading to successful funding that is a deciding factor for achieving career milestones.

### Cultural Differences

International scholars, whose cultural backgrounds are vastly different from Western nations such as the US, sometimes think they will seem too bold, straightforward, or even disrespectful to superiors and authority figures if they speak up without permission or hesitation. Some people misinterpret cultural and language barriers as a lack of confidence or initiative, or as intention to challenge people or propose solutions. Potentially, such misinterpretations can harm one's growth and career. International scholars benefit tremendously when they learn and adapt to Western or American cultures, which encourage innovation, proactive thinking, and self-promotion. In a rapidly evolving research landscape marked by new and emerging technologies, gaining courage to “jump on a moving train” may be critical for scientists from nations where more careful approaches are common or respected. Compared to other nationalities, Americans tend to be goal-oriented, a characteristic that may help speed actions and favor competitive environments. Asian scientists may take a more process-oriented, step-by-step approach that does not necessarily win competitions. Notably, blending new experiences and skills with the strengths they learned at home can strengthen their career prospects.

Having completed their doctoral degrees abroad, most international scientists' first encounter with the American academic system is as a postdoctoral fellow. Thus, they must navigate vastly different academic institutional policies and systems. Foreign scholars, particularly women, are often young adults venturing into family responsibilities, which add to the burden of acclimating to a new country with different norms. From deciphering amenities (e.g., finding good housing, setting up utilities and bank accounts, working out transportation options and schooling for children) to familiarizing themselves with distinctly different work cultures in a completely new environment can overwhelm some international trainees. Moreover, the rigors of academia (e.g., challenging experiments and long hours, working toward multiple publications, and writing successful grants) exponentially compound the challenges faced by international scholars. Some institutions have international societies or postdoctoral associations that can help with setting up some accommodations, but such support groups are relatively rare. Thus, many internationally trained scientists struggle alone. These challenges drive our efforts to share our views with young international scholars.

Beyond postdocs, similarly challenging scenarios confront the larger spectrum of international scholars who enter the academic system in the US, either early as doctoral students or later as faculty members. Regardless of where international scholars begin their journey, early steps to successfully assimilate into the new academic system and way of life can make a large difference. Learning to communicate effectively, think beyond one's limitations, and open up to new opportunities can be very beneficial. Importantly, students must learn to network, be openminded and proactive, and take a multidisciplinary approach to introduce themselves and their work to their new community. International students and postdocs must do their due diligence in finding the right doctoral lab and mentor and seek to match not only the science and academic experiences that interest them, but also the culture of the lab and the mentoring history of the principal investigator (PI). Importantly, she/he should recognize that a mentor–mentee relationship works both ways. Proactive communication helps a PI understand each trainee's goals and makes a mentor–mentee team productive. Since grants are paramount in shaping one's academic career, international scholars should strive to develop their scientific communication skills and grant writing styles. The importance of guiding international scholars to various grant opportunities cannot be stressed enough. Administrative offices or leadership departments may help identify funding opportunities, and grant writing classes can help students write competitive applications for institutional, regional, and national funding.

Navigating visas to maintain legal status to live and work in the US is a significant liability for international scholars. International students begin their US journey with F-1 student visas, then optional practical training (OPT) visas, and finally J-1 (exchange visitors) or H-1B (specialty occupations) visas. Most international postdoctoral researchers have a J-1 visa until they are eligible to apply for a self- or employer-sponsored green card. Some institutions may not support postdocs' H-1B visa or green card applications, adding the pressure on internationally trained scientists who wish to develop careers in the US^12^. Visa restrictions and requirements are complex, expensive, and may risk work–life status, creating stress. Depending on the flexibility and understanding of mentors and institutions that sponsor them, this vulnerability can limit available opportunities. Academic research institutions have administrative services dedicated to international students, postdocs, and faculty members. International scholars should familiarize themselves with such resources and utilize them appropriately for routing daunting, meticulous, and time-sensitive visa transitions.

## Conclusions

Silence is no longer acceptable when social injustice issues become intolerable in our progressive scientific society. The May 2020 murder of George Floyd in Minneapolis, Minnesota, led to worldwide protests of police brutality, racism, and lack of accountability toward African Americans, opening a wide door for an overall review of the issues of social justice and equality beyond racism in the US and around the globe. Attempts to address disparities in health care, education, judicial systems, and the spectrum of workplaces have been an ongoing demand for several decades. Using technology to document violations and social media to spread awareness of prejudices has helped increase awareness in all institutions, including those in the scientific community. This article discussed social justice issues relevant to African- and Asian-Americans, women, and LGBTQ+ as well as challenges faced by international scholars. Importantly, we provide future perspectives to resolve these self-made social determinants (Figure 2). While increased awareness is a critical first step, it is not enough. We must act together, as individuals and as institutions, to introduce real changes that establish and provide inclusive, fair, and safe environments to all. NAVBO's commitment to diversity, equity, and inclusion strongly supports these goals.

## Data Availability Statement

The original contributions presented in the study are included in the article/supplementary material, further inquiries can be directed to the corresponding author/s.

## Author Contributions

MG and MA assembled the materials and coordinated the efforts. All authors contributed to the design and writing of this article.

## Author Disclaimer

Contents of this article do not necessarily represent the official position of NAVBO. The authors of this article represent their own opinions.

## Conflict of Interest

GG was employed by Healthcare and Public Health at Google/YouTube. The remaining authors declare that the research was conducted in the absence of any commercial or financial relationships that could be construed as a potential conflict of interest.

## Publisher's Note

All claims expressed in this article are solely those of the authors and do not necessarily represent those of their affiliated organizations, or those of the publisher, the editors and the reviewers. Any product that may be evaluated in this article, or claim that may be made by its manufacturer, is not guaranteed or endorsed by the publisher.

## Footnotes

^1^ https://www.navbo.org/about-us/policies

^2^ https://www.navbo.org/archive/

^3^ https://www.nytimes.com/2020/05/31/us/george-floyd-investigation.html

^4^ https://www.cnn.com/2021/04/20/us/derek-chauvin-trial-george-floyd-deliberations/index.html

^5^ https://www.washingtonpost.com/national-security/george-floyd-police-officers-federal-charges-civil-rights/2021/05/07/c30fdfb6-ac34-11eb-acd3-24b44a57093a_story.html

^6^ https://economictimes.indiatimes.com/news/international/world-news/us-president-joe-biden-to-america-after-floyd-verdict-we-cant-stop-here/articleshow/82185501.cms?from=mdr

^7^ https://www.nih.gov/ending-structural-racism/unite

^8^ https://www.nsf.gov/statistics/seind14/content/chapter-5/at05-15.pdf

^9^ https://www.science.org/content/article/more-action-needed-retain-women-science

^10^ https://publications.parliament.uk/pa/cm201314/cmselect/cmsctech/701/701.pdf

^11^ http://curt-rice.com/about/

^12^ https://www.ncbi.nlm.nih.gov/books/NBK268781/
